# LGBT + academics’ and PhD students’ experiences of visibility in STEM: more than raising the rainbow flag

**DOI:** 10.1007/s10734-023-00993-2

**Published:** 2023-01-13

**Authors:** Marco Reggiani, Jessica Dawn Gagnon, Rebecca Jane Lunn

**Affiliations:** 1 Department of Civil and Environmental Engineering, University of Strathclyde, Glasgow, UK; 2 Manchester Institute of Education, University of Manchester, Manchester, UK

**Keywords:** STEM, LGBT +, Visibility, Inequalities, Inclusion, Higher education

## Abstract

The experiences of lesbian, gay, bisexual, and transgender (LGBT +) individuals in Science, Technology, Engineering, and Mathematics (STEM) are still understudied and, despite some improvements, are still characterised by patterns of exclusion, disadvantage, and discrimination. In this article, we explore how visibility is perceived and navigated by LGBT + academics and PhD students in STEM, with a focus on the ways that interlocking systems of oppression impact people and groups who are marginalised and historically excluded. This article draws on a broader research project about the experiences of women and LGBT + people in STEM that was conducted between 2019 and 2020 at a UK university and is framed by intersectionality theory. Based on the thematic analysis of interviews and focus groups with 24 LGBT + participants, findings suggest that visibility is still a risk for LGBT + academics and PhD students in STEM. We found that the labour of navigating visibility was perceived as an unfair disadvantage and that the focus on individuals’ visibility in the absence of meaningful and transformative inclusion initiatives by higher education institutions was regarded as tokenistic. The article argues that addressing LGBT + visibility should firstly be an institutional responsibility and not an individual burden and that this work is essential to set the conditions for personal visibility to happen by choice, safely and without retribution.

## Introduction

The last decade in the UK has seen increased effort from higher education (HE) institutions to understand and improve the experiences of lesbian, gay, bisexual, and transgender (LGBT +) individuals^1^ in STEM (Science, Technology, Engineering, and Mathematics). This is a welcome development in fields historically characterised by a lack of equality, diversity, and inclusion and in which individuals belonging to underrepresented and historically excluded groups still face systemic inequalities that affect access, retention, well-being, and career prospects (American Physical Society, 2016; Blackburn, 2017; Cech & Waidzunas, 2021; Institute of Physics et al., 2019; Ireland et al., 2018; Yoder & Mattheis, 2016). Nevertheless, initiatives are limited in scope and usually focused on promoting the visibility of LGBT + students and academics rather than addressing systemic causes of bias and discrimination, which can serve as substitution for actual institutional accountability for equality and inclusion (Ahmed, 2012). Significant differences exist across universities due to their institutional cultures and practices, or the wider contexts in which they operate.

The persistent challenges faced by LGBT + people in STEM are partly due to the fact that existing literature has prioritised understanding and reducing inequalities related to other underrepresented groups, chiefly by gender (Blackburn, 2017). This is an oversight as existing studies have highlighted how sexual minority, transgender, or gender non-conforming individuals face significant barriers to inclusion in STEM including hostility, homophobia, and transphobia (Bilimoria & Stewart, 2009; Cech & Waidzunas, 2011; Freeman, 2020; Mattheis et al., 2019; Yoder & Mattheis, 2016). As a result, LGBT + staff and students suffer from structural disadvantages and discriminations that are rendered invisible not only by heteronormative practices but also by partial and non-intersectional approaches to social inequalities. The solutions offered in terms of policies and practices to these problems are often inadequate and reproduce normative frameworks instead of creating the conditions for LGBT + people to thrive (Lange et al., 2019).

We contribute to addressing this gap by examining the experiences around visibility of LGBT + academics^2^ and PhD students in STEM. In particular, we employ intersectionality theory (Collins, 2015; Crenshaw, 1989) as a framework to investigate how visibility is perceived and navigated by LGBT + individuals, with a focus on the ways “interlocking systems of oppression” impact people and groups who are marginalised and historically excluded (Collins, 1986, p. S20). The current move towards creating more inclusive STEM communities—and the focus on showcasing LGBT + researchers it entails—prompts for further examination into visibility and its consequences, and how disclosure of sexual and/or gender identities is perceived and navigated by LGBT + academics and students.

This article draws on a broader research project about women and LGBT + academics and PhD students in STEM conducted between 2019 and 2020 at a UK university and focuses on LGBT + participants. We found that, for LGBT + people in STEM, navigating visibility is perceived as a burden, tokenistic, and still presents potential risks—including exclusion, harassment, and career setbacks. We conclude the article by arguing that creating the conditions for safe and equitable LGBT + visibility, where all LGBT + people can thrive, should be an institutional responsibility and not an individual burden.

## Literature review

Studies in higher education (HE) focused on STEM disciplines employ a variety of concepts and theories to understand LGBT + experiences and identities, including campus climate, minority stress theory, and queer theory (Bilimoria & Stewart, 2009; Cech & Waidzunas, 2011; Mattheis et al., 2019; Patridge et al., 2014). Researchers have focused on academics, graduate, and undergraduate students, and expanded the field of inquiry from HE to research institutes and STEM organisations (American Physical Society, 2016; Institute of Physics et al., 2019). Nevertheless, prior studies have found it hard to account for the nuanced experiences of the various identities included under the LGBT + umbrella. These gaps are compounded by a lack of intersectional research, by incomplete demographic data, and a semi-exclusive focus on North America or the UK.

### The experiences of LGBT + people in STEM

Previous research suggests that STEM subjects and departments are usually perceived as more hostile to LGBT + people than social sciences and humanities (Bilimoria & Stewart, 2009; Hughes, 2018). Pervasive stereotypes about who gets to be a scientist, a less diverse workforce and student population, and invisibility are frequently mentioned amongst the possible causes (American Physical Society, 2016; Freeman, 2020). The positivist epistemology and the language of STEM further reinforce marginalisation by rendering LGBT + identities and experiences seemingly irrelevant, as if LGBT + people in STEM can take their identities off like a jacket at the laboratory, office, or classroom doors (Cech & Waidzunas, 2011; Linley & Nguyen, 2015). Students and academics in engineering and science operate under the influence of professional cultures that actively prevent discussion of social justice issues and are then less prepared to understand and counteract systemic inequalities (Cech, 2013).

Studies indicate that the persistence of heteronormativity, homophobia, and transphobia in STEM has a significant impact on the levels of personal and professional development of LGBT + faculty and students. In the UK, a 2019 report revealed that “28% of LGBT + respondents stated that they had at some point considered leaving their workplace because of the climate or discrimination”, a percentage that rose to nearly 50% for transgender respondents (Institute of Physics et al., 2019, p. 5). Cech and Waidzunas (2021) found that LGBTQ STEM professionals were more likely to have fewer career opportunities, face professional devaluation, and suffer from social exclusion than their non-LGBTQ colleagues, whereas findings from Nelson et al. (2022) indicate that nondisclosure of queer identities was associated with reduced publications rates for LGBTQA scientists.

It is worth noting that these pressures do not exist in isolation and intersect with other external and internal influences to determine a wide range of individual experiences. Women face pervasive stereotypes and biases in STEM, along with discrimination and exclusion in areas like recruitment, retention, and career progression (Blackburn, 2017; Weeden et al., 2020). People of colour^3^, disabled people, or working-class/people from lower income backgrounds also suffer inequities and barriers to inclusion in STEM (Grineski et al., 2018; Ireland et al., 2018; Sukhai & Mohler, 2016).

### LGBT + visibility

Coming out, outness, and visibility are important constructs used to explain the experiences of LGBT + people that are often employed interchangeably, albeit being underpinned by different theoretical assumptions. For decades, scholars employing a psychological perspective framed coming out as a linear development leading from acceptance of one’s sexual identity to eventual disclosure and integration into society (Carrion & Lock, 1997). Later studies reframed coming out as a dynamic process characterised by continuous becoming, and in which outness is constantly negotiated via strategic tactics of identity management (Klein et al., 2015; Orne, 2011). As research progresses, and with stronger links with post-structural, critical, and intersectionality theory, the discourse around outness is further problematised by the systemic and contextual mechanisms of visibility (Catalano, 2015; Leyva et al., 2022). Similarly to what happens to other oppressed groups (Buchanan & Settles, 2019), visibility, hypervisibility, or invisibility become modes through which LGBT + individuals navigate the asymmetric power relations between hegemonic and marginalised groups.

As one of the paradigms through which we understand LGBT + experiences in HE (Lange et al., 2019; Renn, 2010), visibility presents advantages when it comes to the analysis of inequities. This is because, although there is no universal understanding of visibility as a social category, visibility relates to the ways one is (mis)recognised by others and perceptions are shaped across sites and subjects—thus, it makes explicit the relationship between identities, perception, and power across individuals and groups (Brighenti, 2007; Lewis & Simpson, 2012). Both the literature and LGBT + activism in HE have been historically centred on the experiences of white, cisgender, able-bodied, middle-class individuals (Renn, 2010). By using intersectionality theory (Collins, 2015; Crenshaw, 1989), researchers have started to highlight how the ways visibility is performed and negotiated are compounded by interlocking systems of oppression and privilege—for instance by Black queer students in STEM (Leyva et al., 2022).

These concepts and theories have influenced the ways scholars have looked at LGBT + experiences in HE, although the relationship between scholarship and theory is often not sufficiently explicit (Duran et al., 2022). Research has focused on campus climate and structural issues to investigate factors that might facilitate or hinder visibility (Bilimoria & Stewart, 2009; Cech & Waidzunas, 2011; Ellis, 2009). Results from these studies suggest that coming out and being visible as LGBT + people remain difficult decisions. As discussed by Prock et al. (2019), for some, visibility, active engagement with the LGBT + campus communities, and advocacy are a source of resilience and inspiration. However, these possible benefits come with risks that can have a significant impact on well-being and careers including harassment, tokenisation, and discrimination. As literature is increasingly adopting intersectional frameworks, studies have also started to reveal differences at the intersection between sexuality, gender, race/ethnicity, and other social identities (Duran, 2019; Miller & Downey, 2020; Nicolazzo, 2017).

Findings on how LGBT + people in STEM experience visibility have been contradictory—and sometimes difficult to compare due to the different ways coming out, outness, and visibility are used. In their study, Patridge et al. (2014) found that despite higher levels of outness when compared to non-STEM departments, outness was negatively correlated with comfort. In contrast, the Exploring the Workplace for LGBT + Physical Scientists (2019) report found that “those who described themselves as out to everyone were much more likely to report a comfortable working climate” (p.6). However, results varied across the LGBT + spectrum. Findings from Yoder and Mattheis (2016, p.21) highlight that significant differences existed across STEM fields and that “participants working in STEM fields with better representation of women reported a higher degree of openness”.

Building on and complementing these studies, this article offers further insight into the ways that LGBT + academics and PhD students experience visibility in STEM. By using intersectionality theory and qualitative data collected at a UK university, we do so by drawing attention to the ways these experiences unfold within the systemic inequalities that characterise institutions and STEM fields.

## Theoretical framework

The study is theoretically framed by intersectionality theory (Collins, 2015; Crenshaw, 1989) in recognition of the unique and multiple ways each participant may be socially constructed, and experience oppression and privilege. Originating in the context of Black feminism, the US legal system, and social justice projects focused on the interlocking oppression by race/ethnicity, gender, and social class, intersectionality theory has since expanded across fields of inquiry. While intersectionality theory has become a primary lens for feminist scholarship, this presents theoretical and methodological uncertainties, questions, and paradoxes (Nash, 2008). Intersectional approaches to the study of inequalities enable the inclusion of historically erased voices, value experiential knowledge, and facilitate analytical strategies that engage with the multiple configurations of oppression and privilege (Choo & Ferree, 2010; Moradi & Grzanka, 2017). As critical praxis, intersectionality is a call to action towards transformation and social justice (Collins, 2015).

Our study employs intersectionality theory in non-additive, situated, and expansive ways to capture and interpret the “complexities of compoundedness” that characterise social life (Crenshaw, 1989). When applying intersectionality theory in the context of HE institutions and STEM, our aim is to be counter-hegemonic and contend with flawed ideologies of neutrality, objectivity, and meritocracy that (re)inforce privilege and reify interlocking systems of oppression (Bhopal, 2018; Seron et al., 2018). Our study centres the experiential knowledge of academics and PhD students who are historically excluded. Findings have been used to design transformative initiatives for LGBT + people and other oppressed groups in STEM, advocate for change, and influence policy in our institutions and in the sector. When discussing inclusion, we do so from a “dialogical” and “transgressive” conception as defined by DeLuca (2013) that rejects dualism and normative approaches and embraces complexity, intersectionality, and social justice.

It is important to note that, when discussing the notions of sexual identities and gender identities, we do so from a post-structural perspective (i.e. queer theory). This is because we understand identities as multiple, fractured, unstable, fluid, situated in time and space and historically constructed (Butler, 1990; Sedgwick, 1990). Intersectionality and queer theory benefit from each other to develop scholarship that connects anti-essential and non-normative conceptualisations of identity and critical approaches accounting for the material consequences of intersecting oppression and privilege (Davis, 2008; Fotopoulou, 2013). Using both intersectionality and queer theory lenses is particularly beneficial for an analysis focused on LGBT + visibility. This helps bring to the fore the “performative”, contingent, and problematic qualities of (in)visibility (Benozzo et al., 2015; Butler, 1990; Halberstam, 1998) while examining how interlocking and situated systems of oppression shape the ways individuals and groups are (mis)recognised, controlled, or empowered.

## Methodology

The data included in this article were collected between November 2019 and March 2020 from a series of semi-structured interviews and small focus groups with 82 participants at a UK university who were recruited for a broader research project about women and/or LGBT + academics and PhD students in STEM (or with a STEM background). Participants included 38 academics and 44 PhD students. Amongst them, there were 24 LGBT + individuals whose experiences are the focus of this article. Participants were recruited through existing networks, online, on campus, and snowball sampling.

The research questions leading the broader research project from which data for this article are drawn include the following: (1) What are the systemic challenges women and LGBT + academics and PhD students in STEM face? (2) Which policies and practices are best for fostering inclusivity? (3) How do participants who are women and/or LGBT + people in STEM navigate their educational and career experiences in HE? By doing so, the project seeks not only to better understand experiences of two historically marginalised and underrepresented groups in STEM but also to inform improvement to policies and practices, thus removing barriers to inclusion both at the institution where the research takes place and in the sector. This article focuses on the thematic analysis of the interviews with LGBT + individuals, particularly around four themes related to the experience of visibility.

### Design

In this study, we engage with intersectionality theory from a post-structural perspective. Understanding identities as socially constructed resonates with intersectionality, insofar as research is contextual, reflexive, co-constituted (Choo & Ferree, 2010; Moradi & Grzanka, 2017), and post-structural approaches are key to the challenges that queer theory leverages against essentialist ideas of identity (Butler, 1990; Sedgwick, 1990). Generalisation of results is not the aim of this study. Instead, the qualitative methods that we choose in order to answer our research questions (and the ways we use them) put emphasis on capturing and interpreting the experiences of historically excluded individuals.

In terms of positionality, the co-authors have lived experiences as LGBT + individuals and/or women in higher education (including social sciences and STEM fields). Our understanding of sexuality and gender is uniquely shaped by the shifting intersections of multiple social identities, systems of oppression, and privilege. This both facilitated and limited the ways we could relate to our participants and interpret their experiences. As our study aims to be counter-hegemonic, we engaged in reflexive practices to consider the ways we collected and analysed data while avoiding furthering the status quo, reifying existing norms, or homogenising our participants’ accounts (McDonald, 2013).

### Participants

Amongst the participants on which we focus for this article (*n* = 24), seven were academics and 17 were PhD students. Six identified as bisexual, four as gay woman/lesbian, nine as gay man, and five preferred to self-describe (see Table 1 for an overview of selected participants’ characteristics). Regarding gender, 13 participants identified as women (including one transwoman), 10 as men, and one as non-binary/genderqueer. Fourteen participants described themselves as White British, eight as having other White backgrounds, and two as Asian/Asian British. Nine participants reported having one or more disabilities. Nine participants self-identified as working-class, 14 as middle-class, and one declined to indicate their social class identity.

**Table 1:** Participants’ characteristics

Pseudonym^a^	Gender and sexual identities^b^	Race/ethnicity^c^	Faculty^d^	Career stage^e^
Olivia *^f^	Woman/asexual	White British	Science	PhD student
Ava	Woman/transgender/gay woman/lesbian	White British	Science	PhD student
Emily *	Woman/bisexual	White British	Science	Mid-career
Michael *	Man/gay man	Other White	Engineering	Mid-career
Grace	Woman/bisexual	White British	Science	PhD student
Matthew	Man/gay man	White British	Engineering	PhD student
Paul	Man/gay man	White British	Science	Senior-career
Alba	Woman/hetero-bisexual	Other white	Engineering	PhD student
Maya	Woman/bisexual	White British	Science	PhD student
Thomas	Man/gay man	White British	Engineering	PhD student
Sai *	Man/gay man	Asian or Asian British	Business School^g^	PhD student
Roberta	Woman/gay woman/lesbian	Other White	Engineering	PhD student
Camila	Woman/gay woman/lesbian	Other White	Engineering	Early-career
Chloe *	Woman/bisexual	White British	Business School^g^	PhD student
Vee *	Non-binary/genderqueer/queer	White British	Science	PhD student
Finn	Man/bisexual/gay man	Other white	Engineering	PhD student
Ann	Woman/bisexual	White British	Science	PhD student
Diego	Man/gay man	Other White	Engineering	Mid-career
George *	Man/gay man	White British	Engineering	Early-career
Lewis *	Man/gay man	White British	Engineering	PhD student
Sophia	Woman/gay woman/lesbian	Other White	Engineering	PhD student
Claire *	Woman/questioning/straight-ish	Other White	Engineering	Mid-career
Eric	Man/gay man	Asian or Asian British	Science	PhD student
Lucy	Woman/bisexual	White British	Science	PhD student

^a^Pseudonyms have been assigned by authors

^b^For sexuality, we provided categories as suggested in Pasterny (2016) and also space to self-describe

^c^For race/ethnicity, we followed census categories and also provided space to self-describe

^d^To protect participants' anonymity, only faculty affiliation was collected and/or reported

^e^Academics self-selected their career stage amongst three options (early, mid, or senior career)

^f^The * symbol indicates participants who reported having one or more disabilities

^g^Participants with a STEM background and doing STEM research, albeit not based in a STEM faculty at the time of the study

It is worth noting that, in the sample of participants recruited for the broader research project (*n* = 82), 13.4% (*n* = 11) identified as people of colour, including Black women, Asian/Asian British individuals, and mixed racial/ethnic background individuals. This percentage reflects the underrepresentation/historical exclusion of people of colour, particularly Black individuals, in STEM and across HE in the UK. For example, people of colour make up 12.9% of all academic staff in STEM in the UK, with Black scholars being just 1.6% (Advance HE, 2022, p. 157). The percentage of people of colour included within the broader research project is higher than that of the university where the study takes place—a conscious effort by the research team to include individuals who are oppressed and erased by intersecting systems of oppression (Choo & Ferree, 2010; Moradi & Grzanka, 2017). Similarly, the percentage of participants who identified as disabled (25.6%, *n* = 21) is higher than that reported by the institution and in the sector (Joice & Tetlow, 2021). This possibly highlights the risk of formal disclosure of one or more disabilities in STEM and HE, particularly for disabled people experiencing the impact of multiple systems of oppression (Careers Research & Advisory Centre, 2020; Miller & Downey, 2020).

### Data collection and analysis

During focus groups and interviews, which lasted for approximately one hour each, participants shared their overall experiences in STEM, including those before joining the University. These methods were chosen to explore research questions from participants’ views and produce knowledge via contextual interactions between the researchers and participants (Kvale & Brinkmann, 2009; Wilkinson, 1998). Focus groups and interviews were facilitated by the first and second author who both have significant experience in qualitative methods. The semi-structured interview protocol was flexible and placed equal emphasis on eliciting information on systemic challenges and discrimination and best practices and supportive behaviours, as well as experiences related to navigating sexuality, gender, and other identities. When applying intersectionality to the design of the interviews (Windsong, 2018), we prepared questions that allowed participants to broadly discuss how they identified, what gender, sexuality, and other social identities meant to them, and how these influenced their experiences. We also explicitly prompted participants to reflect on the factors that might contribute to inequalities in their fields, at the institution, and/or in their careers. Twelve participants also engaged with reflective writing to add to or clarify what was shared during the interviews.

After transcribing, checking for accuracy, and fully anonymising transcripts, data were thematically analysed. Our approach to thematic analysis was recursive and reflexive (Braun & Clarke, 2006) while incorporating some elements of template analysis as defined by Brooks et al. (2015) to facilitate collaborative coding. By considering the whole data set and notes taken during the interviews, the first and second author developed an initial set of codes together that included a number of a priori codes that we used tentatively as a starting point for the analysis. These included, for example, systemic challenges/inequalities (e.g. disadvantage; stereotype; prejudice; harassment, bullying, and discrimination) and identities/social categories (e.g. gender identity, sexuality, and race/ethnicity). The same sample of interviews were coded separately by the authors before comparing coding choices. Themes, including those discussed in this article, began to emerge within and across broader ones and an initial codebook was developed and refined over multiple rounds of coding until consensus was reached. Each draft of the codebook was discussed with the third author and the project advisory board, including academics from both STEM and social sciences, to increase the trustworthiness and reliability of the analysis (Nowell et al., 2017).

### Context and limitations

The study is situated in a predominately white university located in a major urban centre in the north of the UK. The institution is active in a wide variety of STEM subjects. While there is a more equitable gender balance amongst PhD students and early-career researchers, the percentage of women in senior roles is much lower, an issue of historical exclusion from senior roles for a number of underrepresented groups that persists across the UK HE sector. At the time data collection was undertaken, there were no other active projects to improve the experiences of LGBT + individuals in STEM, and only a few other initiatives existed at the institutional level around LGBT + inclusion more broadly.

While our participants were all studying and working in the same institution, the interviews and focus groups provided space for them to reflect on their entire experiences in HE as students and staff across multiple institutions, particularly though not only in the UK.

The majority of the participants variously identified as LGB, as it is usually the case for studies focused on the broader category of LGBT + people and in the general population. Nevertheless, our analysis was intended to broadly represent and include a wide spectrum of LGBT + identities despite the difficulty of recruiting, for example, more trans or non-binary individuals. Acknowledging the voices of minority groups within the LGBT + umbrella, which within this article includes one trans woman, one non-binary/genderqueer/queer person, and one asexual person, is key to avoiding homogenising and erasing queer experiences and enables more inclusive, socially just, and meaningful usage of “queer data” (Guyan, 2022; Ruberg & Ruelos, 2020).

Similarly, and while more diversity exists amongst participants of the broader research project where the data for this article originated, given that LGBT + participants identified either as White British, other White backgrounds, or Asian/Asian British, there are limits to our ability to fully discuss the experiences of people of colour.

Many PhD students often work as staff in some capacity, so including both academics and PhD students within this study provides deeper insight into LGBT + in STEM educational and career experiences. Although the majority of our sample is overrepresented by PhD students at various stages of their doctoral studies, by focusing on both academics and PhD students, we recognise and are able to illuminate that challenges for LGBT + people in STEM begin early in academic journeys (LaSala et al., 2008).

The data collected for the study concluded just as the start of the first COVID-19 pandemic lockdown in the UK (March 2020). Therefore, our data do not reflect the possible impact of the pandemic on LGBT + academics and PhD students.

## Results

We articulate results around four themes shaping the participants’ experiences of visibility in STEM. These were the following: perceived lack of diversity, visibility, and representation of LGBT + identities in STEM; navigating the personal and professional impact of visibility; interlocking systems of oppression and visibility; and LGBT + visibility in STEM: an institutional imperative.

### Perceived lack of diversity, visibility, and representation of LGBT + identities in STEM

Regardless of their career stage or their different identities, many participants reported they did not know any other LGBT + staff or students. The lack of visibly out colleagues and peers reinforced the often discomforting impression of isolation in their work environment—a feeling that remains consistently reported in the literature (American Physical Society, 2016) despite the work towards progress on LGBT + inclusion. As Camila, a lesbian woman and early-career academic, who did not come out during her PhD, puts it: “I don’t even know if there are any other [LGBT +] members in my department. I’m sure there must be! But they’re not out”. The number of known individuals was even smaller when considering the intersections between LGBT + identities and other groups traditionally underrepresented in STEM. Even when PhD cohorts included a more diverse range of people, senior roles and leaders seemed to only include people with privileged identities as described by Lewis:

With the PhD students, we’ve got quite a lot of people from all different backgrounds, but the more senior you go, it gets whiter, straighter, more male. The whole top hierarchy is pretty much dominated by [them].

The perceived lack of visibility and representation of LGBT + identities were often discussed by participants as a disadvantage and a consequence of biased and normative professional cultures in STEM that mobilise concepts of neutrality and objectivity to conceal and justify systemic inequalities and privilege (Cech, 2013). Participants reported that discussion around inclusion was almost non-existent in their work environment—and if it was, it was “always [about] women” as reported by Paul, a gay man and a senior academic in science, and never about intersecting axes of oppression other than gender. Others described how their sexual identity seemed to be rendered invisible by heteronormativity, “[they] would immediately assume you’re straight. …To them, that’s normal” said Matthew, a gay, white, and working-class PhD student; a feeling that was felt more acutely by those for whom sexuality was (or was perceived) as the most salient system of oppression.

Similarly to what was suggested by Ellis (2009) while investigating campus climate and the experience of LGBT + students in the UK, feelings of marginalisation were reinforced by the perception that universities were not doing enough to promote awareness, remove barriers to inclusion, or tackle homophobia and transphobia, despite being formally compliant with provisions mandated by anti-discrimination legislation. On the contrary, participants highlighted that, at times, everyday practices and unwritten rules in departments seemed to reinforce heteronormativity and hegemonic masculinity—which other studies have discussed as hypermasculinity and “bro culture” in STEM (Miller & Downey, 2020)—rather than creating an environment where everybody can thrive.

### Navigating the personal and professional impact of visibility

The majority of participants indicated that they were out, if not to everybody, at least to some colleagues or peers. However, when coming out was discussed, many noted that it took time and effort to figure out how to be visible and whether it was safe to do so. For example, Sai described that, even if he came out after arriving in the UK from Southern Asia, the absence of other LGBT + people and/or people of colour in his department made it difficult to bring his whole “self as a person” to work. Sai also discussed experiencing homophobia when trying to find community with fellow students:

I kind of joined the [participant’s nationality students’] society … and started feeling good and I was quite open about my sexuality. … I spoke to someone [fellow student in the society] and … when he found out I was gay, he was like, ‘Why are you gay? You can get a woman’. … I backed out of the conversation and … I distanced myself from the society.

Numerous participants agreed that the experience of visibility did not start or end with coming out. Rather, the choice to be visible—thus to be recognised and, possibly, misrecognised (Brighenti, 2007)—amongst asymmetries of power which manifest through heteronormative, sexist, cisgender, and/or racist biases reappeared with different nuances whenever meeting new people or accepting a post in a different institution. For some, this conundrum triggered stress, trauma, and negative emotions, like in the case of Michael, who was concerned about travelling to or collaborating with an institution located in a country where people face prison or persecution because of their sexuality. Others carefully considered the risks of visibility—particularly participants at the early stages of their careers—as both disclosure, nondisclosure, and the ways LGBT + identities are performed in the workplace present challenges and potential professional setbacks (Cech & Waidzunas, 2021; Nelson et al., 2022). Therefore, and regardless of their degree of outness or self-confidence, the effort put into navigating visibility caused both emotional labour and distress.

A small number of academics and PhD students reported passing, covering, or simply not talking about their sexual and/or gender identity. Camila mentioned that arriving in a different country where she was oblivious of cultural norms had been key in her decision to stay “in the closet” during her PhD. Others, such as Ava, managed their different identities selectively:

I’m out as having a girlfriend, but I'm not necessarily out as trans to everyone in the department. … I feel I could. … It’s just … I don’t feel I should have to, almost.

The normative idea that individuals should be valued only for what they achieved meritocratically (Seron et al., 2018) and stereotype threat (Spencer et al., 2016) convinced some participants to avoid references to their sexual identity. For example, Eric, a PhD student identifying as gay, working-class, and Asian/Asian British, was worried that others might “judge you on your private life and … on all the stereotypes that surround a certain label” rather than your research. Similar concerns were expressed by a few participants identifying as bisexuals due to the fear that, as pointed out by Chloe, there might be “cultural implications placed on somebody who maybe isn’t completely straight or cisgender*.*”

Fear of professional retribution was often motivated or reinforced by participants’ experiences of hostility, lack of support, indifference, or dismissiveness towards their visibility as LGBT + individuals in STEM. As reported by Diego, a mid-career academic from outside the UK, the environment felt “very male” and “primitive” in regard to issues of gender and sexuality, particularly around transgender identities. The absence of LGBT + visible role models and senior leaders added further concerns that being visible might hamper career prospects—a result that echoes the importance of visible LGBT + people in senior or leadership positions discussed by Lee (2022). As George puts it:

I don’t know any outwardly LGBT people who are above me … so I don’t have any role models … it would be really nice … [to see that it] didn’t seem to hamper their employment.

As a result, even participants who reported being more open about their sexual identity were weary of being visible as LGBT + in STEM. This was out of fear they might be discriminated against because of their sexuality. As Lewis described:

[There is always] that wee voice in the back of your head that's like, ‘Is this going to hinder me at some point? Is someone going to prevent me from doing something? Is someone going to think something?’

### Interlocking systems of oppression and visibility

The analysis of participants’ accounts highlights that LGBT + academics and PhD students in STEM experience visibility and oppression as mediated by interlocking systems of oppression. The participants who identified as LGBT + and as people of colour discussed having experienced more harassment and exclusion due to their race/ethnicity than their sexual identities. This shaped their views about what it means to be an LGBT + person of colour in the UK, as noted by Sai: “[recently] I have had more racial incidents than anything to do with my sexual identity. So, I am a bit more focused on that right now”. Others recognised how being White and British, and the privileges both identities impart (Bhopal, 2018), rendered oppression based on their sexuality particularly intense. As Thomas, a White, middle-class, cisgender, gay PhD student in engineering said, “[since I am less exposed to oppression] it hits harder when it does hit”.

Women identifying as LGBT + reported incidents of sexism, and discussed the impact that prejudice and discrimination based on gender have on careers in ways that are consistent with findings on the experiences of straight women in STEM (Blackburn, 2017). Some participants pointed out that, since sexual orientation was not something immediately visible to peers and colleagues, navigating their visibility as LGBT + individuals presented unique challenges and advantages because of the different and overlapping ways sexism and homophobia manifested, interacted, and were perceived in the workplace. As discussed by Roberta, a White PhD student from outside the UK, not mentioning being a lesbian might be an advantage. However, not disclosing her sexuality did not shelter her from the effects of the occasional homophobia (e.g. homophobic jokes amongst peers) that distressed her more intensely if compared to sexism. That was because she could imagine sexism targeting all women in the department—whereas she was the only lesbian amongst the PhD students, at least to her knowledge.

Incidents of transphobia and binarism were also reported, often in the guise of micro-aggressive behaviour or misgendering, as highlighted by Vee, a nonbinary PhD student in science:

[another student] knows that I don’t use binary pronouns, so to my face, he will use no pronouns at all to refer to me. I get misgendered when I’m out of earshot.

Some participants noted how initiatives around gender equality are beneficial to improve the climate in the workplace—albeit these are much more supported by institutions if compared to actions to remove barriers and promote equity for LGBT + individuals, people of colour, and other minorities, which is aligned with findings in existing literature (Bhopal & Henderson, 2021).

The intersection between minority sexual identity and other “invisible” identities further complicates the process of navigating visibility. This was especially true for LGBT + participants who are disabled—which is consistent with findings from prior studies (Miller & Downey, 2020). The story of Olivia, who identifies both as asexual and on the autism spectrum, is exemplary:

[When I have to explain myself in academia] I tend to lead off with autistic more because it’s more making sure I get accommodations. ... If I could choose, I’m not quite sure which way round I’d go because, if you say you’re autistic first, you then play into a whole bag of misconceptions. … If I go, oh yeah, I’m asexual, some people just don’t think you can think for yourself.

Notably, Olivia’s concerns were directed not just towards heterosexual and cisgender colleagues or institutions, but also towards other LGBT + individuals. This is because, and similarly to what expressed by other participants, the LGBT + community was not always perceived as fully inclusive for disabled people and those who identified with minority or less represented queer identities (e.g. asexual, non-binary, or intersex people).

### LGBT + visibility in STEM: an institutional imperative

Overall, participants discussed visibility for LGBT + in STEM in positive terms—a conclusion that seemed often the result of a wider and sustained reflection on their identities and the challenges they faced during their careers. As noted by Paul, a senior-career academic in science, progress has been made—at least in terms of basic recognition of the existence of LGBT + identities and compliance with equality legislation:

There was a pride flag flying in the gardens. I thought, wow that’s really quite amazing. … When I saw that I was thinking back to the late 1980s. … That would never have happened before.

Interviewees discussed the possible benefits of visibility from different angles. Some articulated how being out was key to expressing their authentic self. For others, visibility was instrumental to overcoming the isolation they felt as LGBT + individuals in STEM, creating connections, and sustaining their careers. Similarly to what is reported by Mattheis et al. (2019), a few participants described visibility as a strategic choice to “take some action” and actively work toward more equitable, diverse, and inclusive STEM communities—a decision that was sometimes accompanied by a reflection on privilege or solidarity with other marginalised groups.

Although there seemed to be an agreement around the idea that visibility and representation might be a step toward positive change, some participants had reservations about their own visibility. This echoes contradictions and ambiguities of visibility as a social category, particularly of LGBT + visibility in the context of heteronormative and oppressive institutions (Benozzo et al., 2015). In Matthew’s words from his reflecting writing:

I personally don’t feel like we should have to tell people, as this immediately makes you seem *different*. However, if we don’t do this, then how can we empower others and create visibly welcoming, inclusive and diverse environments? This is the one point I struggle to reconcile with.

Some academics and PhD students felt uneasy discussing their sexuality in the context of an increasingly international HE landscape, with, for example, expectations to possibly travel to countries with laws that put LGBT + peoples’ lives in danger. Lack of support, allyship, and opportunities for training around LGBT + issues emphasised the idea that, if made visible, their identities might be misunderstood and suggested that the ways LGBT + visibility was celebrated in STEM and HE were tokenistic. Despite universities raising rainbow flags or posting on social media with calls for the celebration and visibility of LGBT + people during specific times of the year, such as Pride Month, LGBT + History month, or Trans Day of Remembrance, there is in fact very little evidence of meaningful and measurable institutional accountability for creating campus communities where the choice to be visible is made without the risk of discrimination.

In weighing the benefits and challenges of visibility in STEM, participants seemed to favour an approach through which their LGBT + identities were signalled in subtle and informal ways, enabling a less tokenistic experience of visibility. This could happen in everyday conversations, for example, just talking about their partners using same-gender pronouns. A variety of items—such as pins, lanyards, or desk props—were also used as ways to visually identify as members of the LGBT + community, a result that supports similar findings from Lee (2022). These objects often incorporated the rainbow as the mainstream icon of pride. Other times, colours and symbols related to particular queer identities (e.g. the colours of the bisexual or the asexual pride flags) that could be more easily identified by those who “know where to look” for clues about LGBT + identities. As highlighted by Olivia, this was a conscious decision to balance authenticity against safeguarding oneself from prejudice and discrimination—which, it is worth noting, at times might come from other LGBT + individuals:

Part of the reason I specifically wear this wristband is [that] … if people recognize this, they normally have enough knowledge [about less known LGBT+ identities] and it helps.

Most of all, what many participants discussed was a desire for institutions to do more to be accountable for the inclusion that they claim to value through policies and practices that are transparent and measurable, enabling all LGBT + people to thrive.

## Discussion

In this article, we examined interviews and focus groups with 24 LGBT + academics and PhD students in STEM at a UK university to explore participants’ experiences of visibility. Our investigation confirms that the ways LGBT + people in STEM navigate visibility are highly personal, therefore characterised by ambiguities as exemplified by prior research (Institute of Physics et al., 2019; Patridge et al., 2014; Yoder & Mattheis, 2016).

The analysis of the four themes presented in this article shows how visibility is shaped by multiple and intersecting oppression and systemic inequalities. As a result of professional cultures where invisibility and exclusion of a number of identity categories have been the norm (Cech & Waidzunas, 2011), and in universities where initiatives to address LGBT + issues have been limited, STEM faculties and departments were not necessarily seen as welcoming or more inclusive compared to industry or other settings. This contrasts with previous studies finding that LGBT + participants across subject areas rated their academic institution as particularly welcoming (Lee, 2022), or the firmly-held belief that universities are progressive. As our study suggests, the HE landscape in the UK is more nuanced. While some institutions might be faring better in terms of LGBT + equality and inclusion, more than ten years from her original study, we continue to agree with Ellis (2009) that so much more still needs to be done.

As expected, in the data, we found a mix of both affirming and challenging experiences of visibility: although some described disclosing their identities as a way to be their authentic self, create connections, or take action to foster LGBT + equity and inclusion, visibility exposed others to harassment and discrimination. In contrast with Yoder and Mattheis (2016), we did not find significant differences in openness due to the age of our participants. Contrary to our expectations, PhD students and early-career academics were not necessarily more comfortable with visibility when compared to mid-career or senior academics. This might be because of the normative values shaping STEM fields, the lack of role models, and the lack of diversity and inclusion in leadership positions suggested that being visible as LGBT + might be risky and hamper career prospects.

Despite individual differences, and although they expressed the desire for more diversity and representation in STEM, participants usually perceived the work of navigating visibility as an unfair burden for LGBT + individuals. Both academics and PhD students recognised that this challenge is a consequence of biased working cultures and educational environments, where heteronormativity, homophobia, and transphobia are still widespread and have far-reaching consequences (American Physical Society, 2016; Freeman, 2020; Institute of Physics et al., 2019). Yet, when compared to other challenges such as hostility and harassment, the invisible labour of navigating outness and visibility often goes unnoticed and unaddressed. As a consequence, LGBT + people in STEM are placed at a further disadvantage regarding, for example, research opportunities or career progression—a result that might suggests parallels with the experiences of visibility related to other marginalised and oppressed social identities that are not immediately visible, such as being disabled and/or from a working-class background.

Our analysis shows that the labour of visibility that LGBT + people in STEM undertake—an effort that, as others have shown (Bilimoria & Stewart, 2009; Cech & Waidzunas, 2011), is both emotional and performative, and has an impact on both sense of self and STEM identities (Mattheis et al., 2019)—becomes even more complicated when it intersects with multiple axes of oppression. Participants who are both LGBT + and people of colour reported more incidents of racism compared to homophobia; thus, race/ethnicity affected their experiences equally or more intensely than sexuality—a finding that resonates with other studies of HE (Leyva et al., 2022) and contrasts, for example, with the experience of White, cisgender participants in our sample. Additionally, a number of PhD students and academics, particularly those belonging to minority and underrepresented groups under the LGBT + umbrella, discussed how normative expectations around gender identity, gender expression, and ableism reinforce oppression based on sexuality and the ways they had to navigate their careers.

Finally, while contributing to the existing conversation around LGBT + disparities in STEM, this article highlights visibility as something more than just an individual concern or an issue that can be addressed via rainbow props or symbols—although both individual circumstances and signs were important in mediating the experiences of visibility of our participants. Rather, the struggle around visibility reveals the norms and the mechanisms through which power and value are reproduced. These are negotiated in complex and creative ways by LGBT + academics and PhD students in STEM. However, these efforts alone are insufficient to protect them from the risks of visibility in fields and institutions where inequities, hostility, and harassment remain present.

## Conclusion and implications

Through the lenses of intersectionality theory and queer theory, this article expands the understanding of LGBT + experiences in STEM by exploring visibility at the intersection of interlocking systems of oppression, privilege, professional cultures, and institutional practices. We found that visibility in heteronormative institutions can be a risk and is perceived as an unfair disadvantage by LGBT + academics and PhD students. The labour and the pressures of navigating visibility adds to other challenges and inequalities discussed by prior research. The focus on individuals’ visibility in the absence of meaningful and transformative inclusion initiatives by HE institutions was perceived by our participants as tokenistic. On the backdrop of the current push towards inclusion and representation of LGBT + academics and students in STEM, our results carry significant implications for research and practice.

We argue that LGBT + inclusion must be an institutional imperative, rather than an individual burden. This is because, without institutional approaches to equality that are intersectional, multiple and compounded discriminations can be rendered invisible (Crenshaw, 1989; Duran, 2019). The onus is then on individuals to do the work of supposedly creating greater inclusion, or token individuals whose visibility is misrepresented as a sign of inclusivity for all (Ahmed, 2012; LaSala et al., 2008; Prock et al., 2019). However, LGBT + individuals do not owe their institutions visibility or outness, and many studies, including our own, have shown that being out in STEM is still a risk that may not be worth taking. Instead, higher education institutions should focus on creating work and education environments where LGBT + people can thrive. This work is essential to set the conditions for personal visibility to happen by choice, safely and without retribution.

More research and investments are needed to explore both visibility and the experience of LGBT + people in STEM—which remains understudied. A good point to start would be to gather more data to understand the composition and the experience of faculty and student bodies in STEM departments and research institutions. We would like to encourage researchers and practitioners to adopt intersectional approaches to be contextual, inclusive, and mindful of the nuanced interactions between systems of oppression and social identities. Comparative and interdisciplinary studies would be particularly welcome to address this topic from a variety of theoretical perspectives and expand the conversation beyond traditionally siloed academic fields. We believe this research to be essential to bring forward more diverse, equitable, and inclusive STEM communities in HE and beyond.

## Data Availability

Limited, anonymised data can be made available upon valid request after the completion of the project.

## Footnotes

1 In the study, we employ the acronym LGBT + as an umbrella term to describe a wide range of sexual and gender identities in an inclusive and intersectional way. In doing so, we are aware of the critical debate around the usage of blanket labels to categorise and describe identities. When presenting results from other studies we honour the terminology employed by each author.

2 In the article we use the term academics to indicate researchers, teaching staff, and faculty members.

3 In the article, we use the term people of colour, but we also recognise the criticisms of the use of collective terms for underrepresented and marginalised groups and that identity categories drawn from standardised sources (census questions, for example) do not fully reflect the diversity of possible identities. We recognise that terms like people of colour can serve to homogenise the experiences of racism and prejudice in ways that ignore or misrepresent the disproportionate ways discrimination and oppression specifically impact individual communities of colour.
